# Changes in the labor share of enterprises under the green and low-carbon transition: Empirical evidence from China’s carbon emission trading pilot

**DOI:** 10.1371/journal.pone.0300595

**Published:** 2024-04-04

**Authors:** Xin Luo, Jianti Li, Dawei Feng

**Affiliations:** 1 Law and Business School, ShangRao Normal University, ShangRao, China; 2 Guangzhou University School of Economics and Statistics, Guangzhou, China; 3 Institute of Industrial Economics, Jiangxi University of Finance and Economics, Nanchang, China; Inner Mongolia University, CHINA

## Abstract

Green and low carbon reflect the high-quality development, while income distribution is an indicator of the balance of development. Is there a lack of fairness in the process of green and low carbon transition of enterprises? Using data from A-share listed companies from 2009 to 2016, this paper constructs a DID identification framework for controlling the endogeneity problem using the 2013 carbon trading policy pilot as a quasi-natural experiment to empirically test the impact of corporate low-carbon transformation on corporate labor income share in the context of carbon trading policy. The findings indicate that carbon trading policy decreases the labor income share of firms. In addition, we demonstrate that the low-carbon transition promotes labor productivity, suggesting that the Porter’s hypothesis is confirmed in China, but the increase in labor wages is not in tandem with productivity growth, resulting in reduced labor income share. Heterogeneity analysis shows that the impact of carbon trading policy on labor income share is mainly pronounced in larger firms, high technology firms and persistent incumbent firms. Collectively, these results are expected to accurately improve our understanding on the impact of low-carbon transformation of enterprises on income distribution and provide reference for the government to formulate industrial policies and distribution mechanisms under low-carbon economy.

## Introduction

In the context of the proposed national major strategic goals of carbon peaking and carbon neutral, efforts to promote the green and low-carbon transformation of enterprises have been intensified for China to achieve the strategic goal of ecological civilization construction. To achieve low-carbon transformation of enterprises with lower economic costs and to promote compatibility between economic growth development and environmental quality, China officially launched carbon emissions trading pilot in 2013 (Abbreviated "Carbon Trading Policy"). Carbon trading policy has become an effective approach to reduce China’s greenhouse gas emissions and combat climate change. In addition, the green and low-carbon transformation triggered by the carbon trading policy has brought profound economic and social systemic changes, which will certainly have an impact and influence on many fields such as investment, production, circulation and consumption [1]. In this systemic change, companies are expected to optimize their workforce structure and promote technological innovation. An important and much-needed question, then, is whether China’s investment in green technologies and equipment in promoting a green and low-carbon transition will hit the share of labor income? Will it cause an imbalance in the distribution of labor income. There is little discussion on this question in existing studies. Notably, the green and low-carbon transformation of enterprises will accrue productivity gains due to technological upgrading [2–5], but only 20% of the productivity gains will be passed on to wages [6], causing a mismatch between labor compensation growth and productivity growth, and this will reduce the share of labor income. Enterprises’ green and low-carbon transition may incur higher costs, which may directly lower labor wages, and hence affect labor income shares. Green and low-carbon transformation is a manifestation of high-quality economic and social development, while the share of labor income reflects the balance in the social development, and is the key to promoting common prosperity. "*The Outline of the 14th Five-Year Plan and 2035 Vision for National Economic and Social Development of the People’s Republic of China*" states that the principle of distribution should be adhered to: "the growth of residents’ income should be basically synchronized with economic growth, the increase of labor remuneration should be basically synchronized with the increase of labor productivity, and the proportion of labor remuneration in the initial distribution should be increased". In this context, an in-depth exploration of the mechanism linking enterprises’ green and low-carbon transformation to the labor income share may expand our understanding on the impact of enterprises’ low-carbon transformation on income distribution and provide reference data for formulating industrial policies and distribution mechanisms by governments under a low-carbon economy context.

### Against this background, we study the impact of green and low-carbon transformation of enterprises on labor income share under carbon trading policy and its mechanism of action. Compared with the existing literature, this paper has the following main innovative aspects

(1) An in-depth examination of the impact of the low-carbon transition of firms on the share of labor income at the firm level is conducted. Based on the available literature, this is the first study using microdata to test this proposition, which complements and enriches the economic effects in the context of green low-carbon transformation of enterprises and provides a basis for a comprehensive understanding of the implementation effects of low-carbon transformation of enterprises. (2) In-depth analysis is performed on the mechanism of action of the impact of green low-carbon transformation of enterprises on labor income share and verification of the mismatch between labor income growth and productivity growth under green low-carbon transformation of enterprises. To further examine the complexity of the impact of firms’ low-carbon transition on labor income share, differences in the impact of the policy on labor income share across firm size, technology level, and firm persistence are explored. This study will expand our theoretical understanding of the socio-economic impact of the low-carbon transition. Few studies have examined the impact of green low-carbon transition on labor income share under carbon trading policy.

## Literature review and research hypothesis

### Literature review

The concept of carbon trading originated from the concept of emissions trading proposed by economists in the 1990s. This concept refers to the act of enterprises trading emission reduction targets allocated by policymakers in the market. China’s carbon emissions trading system started with the "*Decision of the State Council on Accelerating the Cultivation and Development of Strategic Emerging Industries*" issued in 2010. In October 2011, the National Development and Reform Commission issued the "*Notice on the Pilot Work of Carbon Emissions Trading*", which approved the pilot work of carbon emissions trading in seven provinces and cities, namely Beijing, Tianjin, Shanghai, Chongqing, Hubei, Guangdong and Shenzhen. Since 2013, seven pilot carbon markets have engaged in online trading, covering nearly 3,000 key emission units in more than 20 industries such as electricity, steel and cement. In terms of carbon trading volume, from 2013 to June 2021, China completed 241,309,100 tons of carbon trading, with the market reaching a trading peak of 49,031,000 tons in 2017. The implementation of carbon emissions trading has significantly reduced greenhouse gas emissions by enterprises, forced enterprises to adopt green and low-carbon transformation, and this has improved enterprise production efficiency. The "Porter hypothesis" has been effectively verified in China. However, the question arises as to whether the share of labor income in China’s enterprises also increases along with their productivity when carbon trading policies "push" enterprises to transform and increase their productivity. This question has not been sufficiently discussed in the existing literature.

Current research on carbon trading policy has mainly focused on the effects on regional industrial structure upgrading [7], enterprise R&D innovation [8–10], enterprise investment efficiency [11], short-term value of enterprises [12], and total factor productivity [2–5]. In terms of employment, Wang and Ge [1] evaluated the impact of carbon trading policies on employment by using the micro-data of listed companies from 2007 to 2019 and the progressive differential model, and found that the pilot policies of low-carbon cities significantly improved the employment level of enterprises on the whole. Compared with enterprises in non-pilot cities, the implementation of this policy has increased the employment of enterprises in pilot cities by about 5.11% on average. Liu and Zhang [9] took the carbon emission trading pilot in China as a natural experiment and combined with the data of China’s A-share listed companies, empirically-tested the impact of carbon emission trading system on enterprise R&D innovation from the micro level, and found that the carbon emission trading pilot policy can improve the R&D investment intensity of the enterprises in the treatment group. To encourage more enterprises to be willing to carry out R&D and innovation activities, however, the carbon emission trading pilot policy only has a significant positive effect on the innovation input of large-scale enterprises, but has no significant impact on the R&D and innovation of small-scale enterprises. Shen and Huang [12] made use of the data of listed companies in China and their respective regions from 2006 to 2017, and used the differencing method to find that carbon trading has significant negative impacts on green total factor productivity and enterprise total factor productivity. Carbon trading promotes carbon emission reduction, leading to an increase in operating costs and a decrease in labor productivity, but at the same time, it also has a significant positive impact on capital productivity and promotes the growth of corporate profits and income. In terms of income distribution, Huang and Xu [13] found that the rate of change of labor income share depends on three factors: the size of multiplier effect, the speed of capital deepening, and the size of labor (or capital) saving technological progress. Fan and Li [14] used the "Green Credit Guidelines" issued by China Banking Regulatory Commission in 2012 as a natural experiment to build a Difference-in-Differences model based on the micro-data of China’s listed companies from 2009 to 2018 to evaluate the impact of green credit policies on the labor income share of heavily polluting enterprises. It is found that the implementation of green credit policy significantly reduces the labor income share of heavily polluting enterprises. The above studies provide a solid foundation for us to study the impact of carbon trading system on labor income share. However, few studies have examined the impact of green low-carbon transition under carbon trading policy on labor income share.

### Research hypothesis

According to Clark’s marginal productivity wage theory and Marshall’s equilibrium wage theory, the main factors affecting the share of labor income are the value of the marginal product of labor and the real wage. In this section, we analyze how carbon trading policies affect the labor income share of firms from both marginal productivity and real wage level perspectives.

### Impact of carbon trading policies on firm productivity

The rising productivity of firms is one of the main reasons for the decline in the share of labor income [13, 15–17]. Akerman et al. [6] found that only 20% of the increase in workers’ marginal productivity is transmitted to wages. Therefore, when a firm’s productivity growth exceeds the growth rate of workers’ real wages, the firm’s labor income share will decline [18]. According to Porter’s hypothesis, appropriate environmental regulations can improve the technological level of production and product competitiveness of enterprises [8], which in turn influences productivity. Therefore, we argue that carbon trading policies, as flexible and relaxed market-based environmental regulations, can significantly increase a firm’s productivity, thereby decreasing labor income share. Existing studies have extensively investigated environmental regulation and firm technological progress and firm productivity improvement [19–24]. Among them, Ren et al. [19] directly examined the impact of the emissions trading mechanism on the total factor productivity of enterprises and found that the emissions trading system can promote the total factor productivity of enterprises in two ways: technological innovation and improvement of resource allocation efficiency of enterprises. In terms of carbon trading policies, Wang and Wang [21] reported that carbon trading policies can potentially improve innovation investment and innovation efficiency of firms to boost their productivity based on data from Chinese listed companies from 2008 to 2019. A study by He [24] shows that carbon trading policies increase the willingness of firms to invest in high-skilled human capital to achieve technological progress. Combined with the above studies, this paper argues that the implementation of carbon trading policies will accelerate the growth of firms’ labor productivity more effectively than wage growth thus resulting in a lower share of labor income.

### Impact of carbon trading policy on real wages

With regard to the carbon trading mechanism, each pilot region first sets its own total carbon emissions, and then allocates carbon emission allowances to enterprises. If an enterprise’s actual carbon emissions exceed the value of carbon emission allowances, it has to purchase the corresponding carbon allowances for the excess portion, otherwise it will face severe administrative penalties. This undoubtedly increases the cost of production for companies with high carbon emissions. Firms have an incentive to pass on costs to workers to reduce costs, causing a decline in workers’ real wage income and resulting in a lower share of labor income. Companies with lower actual carbon emissions can sell the surplus carbon emission allowances in the market to attract some gains, which increases labor income and the share of labor income. Using the proceeds gained, firms can hire highly skilled labor [1], leading to further increase in firm productivity levels. Similarly, firm labor income growth does not decrease labor income share in tandem with firm productivity growth. In summary, whether carbon trading policies affect changes in labor income shares due to changes in workers’ wage income should be further tested empirically.

## Research design

### Sample and data

This paper uses A-share listed companies in China’s Shenzhen and Shanghai markets from 2009 to 2016 as the research sample. Firm-level data are mainly obtained from Wind and CSMAR databases (https://www.gtarsc.com/ and https://www.wind.com.cn/). To more effectively estimate the change in the share of labor income under the green low-carbon transition of firms, the following treatments are applied to the sample in this paper. (1) Exclusion of the sample of enterprises that have appeared in ST and *ST. (2) Exclusion of companies that do not meet basic accounting standards. (3) To avoid bias of extreme values on the estimation results, continuous variables are scaled down by 1% and 99% in this paper. After such screening, 2593 companies and 17302 samples are included in this paper.

### Model setting

The carbon emissions trading pilot implemented in 2013 is used as a quasi-natural experiment to examine changes in the share of labor income under the green and low-carbon transition of firms after the implementation of the carbon emissions trading policy using Difference-in-Differences (DID), and construct a double difference model as follows.

$${\mathrm{LS}}_{\mathrm{jt}}=\alpha_{0}+\beta_{1}{\mathrm{did}}_{\mathrm{jt}}+\gamma_{1}X_{\mathrm{jt}}+\delta_{j}+\theta_{t}+\varepsilon_{\mathrm{jt}}$$ (1)

where *LS*_*jt*_ is the explanatory variable of this paper and is the labor income share of firm j in period t. *did*_*jt*_ is the core explanatory variable in this paper, and is the interaction term between the dummy variable Treat_i_ and Time_*t*_, indicates whether province i is approved as a carbon trading pilot in period t. *X*_*jt*_ is a firm-level control variable. *δ*_*j*_ is a firm fixed effect, *θ*_*t*_ is a time fixed effect, and *ε*_*jt*_ is a random error term. The coefficient *β*_1_ indicates the net effect of carbon trading rights on the labor income share of enterprises. When the coefficient *β*_1_ is significantly less than 0, carbon trading policy reduces the labor income share of enterprises, and when the coefficient *β*_1_ exceeds 0, carbon trading policy increases the labor income share of enterprises.

### Variable definition

#### Explained variables

According to studies by Shi et al. [25] and Wang and Huang [26], this paper uses the cash paid by the firm for employees in the current period divided by the total operating revenue to measure the labor income share (LS). Meanwhile, the share of labor income = employee compensation/(operating revenue—operating cost + employee compensation + fixed asset depreciation) is used to calculate the firm’s share of labor income (LS2) as a robustness indicator, drawing from Du et al. [27] and Wang et al. [28].

*Carbon emissions trading pilot policy*. The dummy variable which did to indicate that did = Treat*Time is used in this paper. Where Time is a year dummy variable, Time = 1 when the year is greater than or equal to 2013, otherwise Time = 0. Treat is a dummy variable for the pilot region of carbon trading, Treat = 1 when it is a pilot region and Treat = 0 otherwise. Since Shenzhen is under Guangdong, the treatment group of this paper includes Beijing, Tianjin, Shanghai, Chongqing, Guangdong, Hubei and Fujian. In addition, 7 provinces and cities, and 25 provinces are used as the control group. Besides the impact of carbon trading policy implementation on firms’ labor income share, several factors at the firm level can also affect the change of firms’ labor income share, therefore, the following control variables are selected. (1) Enterprise size (Size), Total assets at the end of the period (yuan) and take the logarithm to express. (2) The year when the company was listed (Age), subtract the year of listing from the current year and take the logarithm. (3) The nature of the enterprise (Soe), private enterprises take 1, state-owned enterprises take 0. (4) Return on assets (Roa), net profit after tax / total assets at the end of the period. (5) Capital structure (Lev), The company’s total liabilities at the end of the period / total assets at the end of the period. (6) Capital intensity (Capital), The company’s total assets at the end of the period / total revenue at the end of the period. (7) Cash flow from operating activities (Cflow), The ratio of net cash flow from operating activities to total assets for the period. (8) Cash holdings (Cash), Cash holdings = (monetary funds + financial assets held for trading)/total assets. Descriptive statistics of the main variables are presented in **Table 1**.

**Table 1:** Descriptive statistics.

Variable	N	Mean	SD	Min	Max
LS	17520	0.121	0.0908	0.00146	0.593
LS2	17520	0.38	0.21	0.01	0.77
lntfp_lp	17520	7.96	1.036	4.71	12.35
lntfp_op	17520	3.59	0.75	0.01	8.07
Size	17520	21.96	1.331	15.58	28.51
Age	17520	1.996	0.911	0	3.296
Soe	17520	0.163	0.370	0	1
Roa	17520	0.0484	0.827	-0.274	108.4
Lev	17520	0.439	0.286	0.055	10.08
Capital	17520	3.016	13.79	0.0945	1039
Cflow	17520	0.0427	0.114	-10.22	0.876
Cash	17520	0.208	0.160	0.01	1.000

## Results and discussion

### Baseline results

This paper tests the change in labor income share under low carbon transition of firms according to model (1), and the results are presented in Table 2. Column (1) presents the result without the inclusion of firm-level control variables, with a coefficient of about -0.005, which is significantly negative at the 5% level, indicating that firms significantly reduce their labor income share during low carbon transition process. Column (2) shows that, after controlling for firm-level characteristic variables, the regression coefficient of -0.004 for did, which is significantly negative at the 1% level, further suggesting that firms significantly reduce their labor income share in the low-carbon transition process. In terms of economic significance, in column (2), for example, the average labor income share in the pilot areas of carbon trading is reduced by about 3.3% on average (= -0.004/0.121*100%) compared to the non-pilot areas. These results suggest that after the implementation of carbon trading policy, enterprises reduce the share of labor income.

**Table 2:** Baseline regression results.

	(1)	(2)
VARIABLES	LS	LS
did	-0.005**	-0.004***
	(-2.55)	(-2.59)
Size		-0.025***
		(-11.19)
Age		0.010***
		(5.33)
Soe		0.003**
		(2.40)
Lev		-0.004
		(-0.50)
Roa		-0.007***
		(-8.03)
Capital		0.001**
		(1.99)
Cflow		-0.063***
		(-7.90)
Cash		-0.007
		(-1.10)
Constant	0.112***	0.655***
	(266.75)	(13.57)
Observations	17,298	17,298
Adjusted R-2	0.764	0.795
Individual fixed effects	Yes	Yes
Time Fix effect	Yes	Yes

Note: Robust standard errors are reported in parentheses.

***, **, and * denote significance at the 1%, 5%, and 10% levels, respectively.

### Robustness tests

#### Parallel trend test

**Fig 1:**
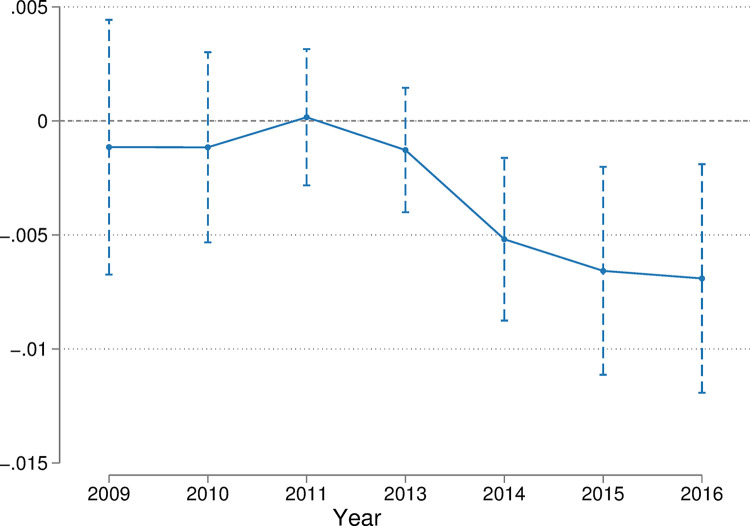
Parallel trend test.

One of the necessary conditions for the Difference-in-Differences model is to satisfy that the control and treatment groups have a common trend of change. Therefore, before the implementation of the carbon trading policy, the labor income shares in the pilot and non-pilot regions tended to be highly similar. Drawing on the event study method proposed by Jacobson et al. [29] for parallel trend testing, this paper uses the year prior to policy implementation as the base year. The parallel trend results shown in Fig 1 indicate that the coefficient estimates for each period before the implementation of the carbon trading policy are not significant. This illustrates that there is no significant difference between pilot and non-pilot regions before the implementation of carbon trading policy, and the study sample passed the parallel trend test. After the implementation of the carbon trading policy, the policy became apparent in the year 2014. This is because, except for Shenzhen, which was the first region to launch the carbon trading pilot in June 2013, the other six pilot provinces started their formal carbon trading pilot in November and December 2013 as well as in 2014. In summary, the test results show high stability.

#### Placebo test

**Fig 2:**
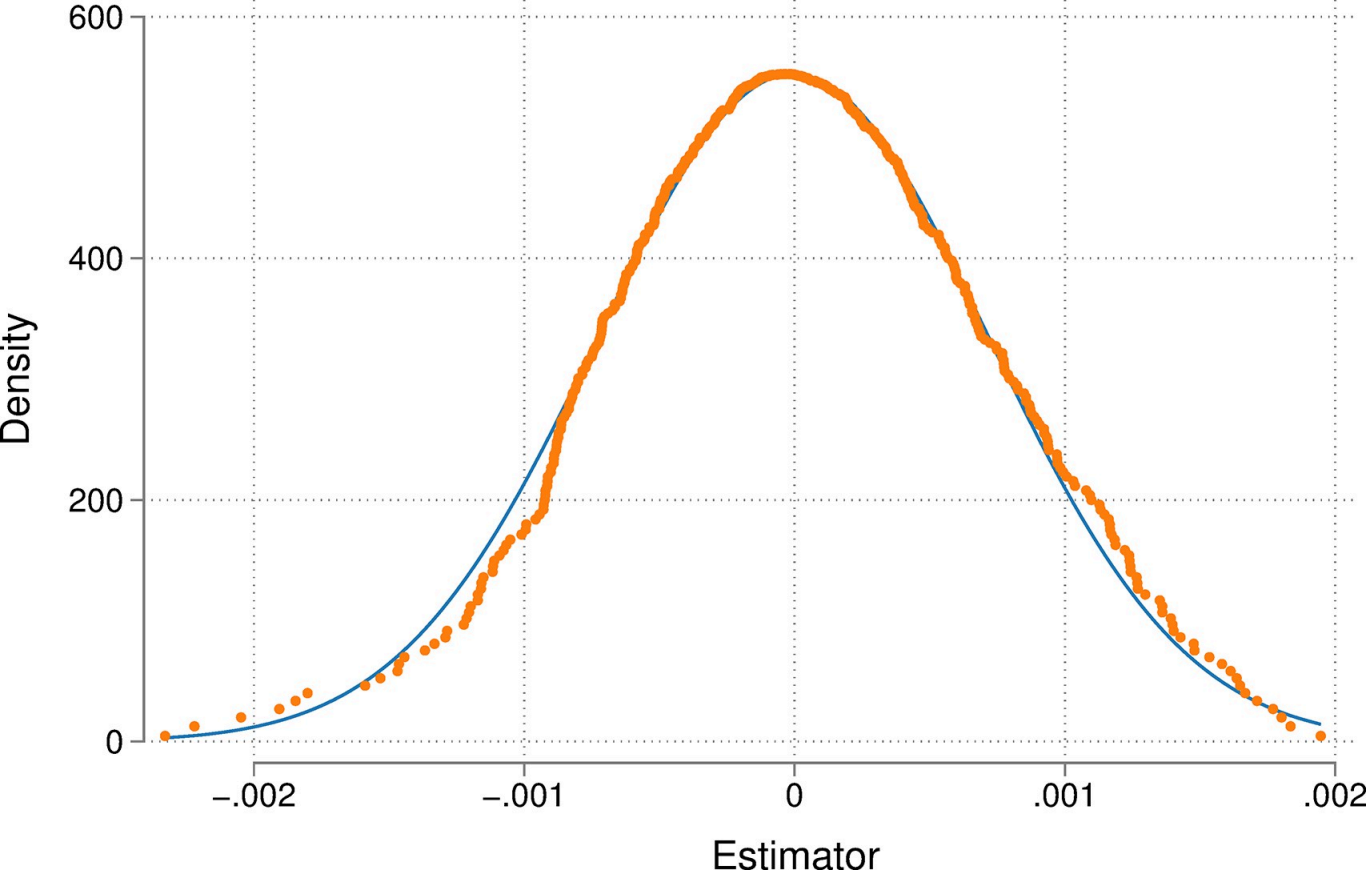
Placebo test.

To further ensure that the regression results are valid and to avoid the effects of unobservable omitted variables on baseline results, a placebo test was conducted by replacing treatment group areas, drawing on Cai et al. [30]. Seven regions were randomly selected as the sham treatment group and the remaining regions were assigned to the sham control group, and the above process was repeated 500 times to obtain the estimated coefficients of the impact of implementing a regional placebo for the pilot carbon trading policy on firms’ labor income share. Fig 2 shows the kernel density distribution of the estimated coefficients of the placebo test. We infer that the estimated coefficients *β*_1_ are concentrated around 0, indicating that the other observed factors hardly affect the estimation results of this paper.

#### PSM-DID

To better select the control group and mitigate the endogeneity problem caused by sample selection bias, the Propensity Score Matching Difference-in-Differences model (PSM-DID) is used to test causality of the effect of carbon trading policies on labor income shares, all else being equal. After using the nearest neighbor matching method, the results shown in column (1) of Table 3 are obtained, and the estimated coefficient did is significantly negative at the 1% level. This further illustrates that implementation of carbon trading policies by low-carbon transition of firms leads to a lower share of labor income.

**Table 3:** Robustness tests.

	(1)	(2)	(3)	(4)
	PSM-DID	Change variables	Change variables	Exclusion of other policies
VARIABLES	LS	LS2	LS2	LS
did	-0.007***	-0.010***	-0.011***	-0.005***
				(-2.97)
Period*Post				-0.006***
	(-3.54)	(-2.89)	(-3.40)	(-3.20)
Constant	0.587***	0.277***	1.265***	0.667***
	(10.78)	(329.69)	(15.78)	(13.71)
Observations	8,773	17,298	17,298	17,302
Adjusted R-squared	0.793	0.681	0.728	0.791
Individual fixed effects	Yes	Yes	Yes	Yes
Time Fix effect	Yes	Yes	Yes	Yes
Control variables	Yes	NO	Yes	Yes

Note: Clustered robust standard errors are reported in parentheses below the coefficients.

***, **, and * denote significance at the 1%, 5%, and 10% levels, respectively. Control variables are estimated as in the Table 2 benchmark.

#### Replacing the explanatory variable

This paper draws on Du et al. [27] and Wang et al. [28] to calculate the firm’s labor income share (LS2) as a robustness indicator using labor income share = employee compensation/(operating revenue—operating cost + employee compensation + fixed asset depreciation). By replacing the explanatory variables and rerunning the regression, the results show in column (2)(3) of Table 2 are obtained, where the estimated coefficient did is significantly negative when no firm-level control variables are included. Column (3) indicates that after adding firm-level control variables, and the coefficients are also significantly negative. These results further suggest that implementation of carbon trading policies by low-carbon transition of firms leads to a lower labor income share.

#### Exclude other policy interference

This paper finds that *the Green Credit Guidelines* during the sample period may interfere with the results of this paper in light of the existing documents. Drawing on Fan and Li [14], this paper includes the interaction term of Period*Post in the benchmark regression. Where Period is a year dummy variable, Period = 1 when the year is greater than or equal to 2012, otherwise Period = 0. Post is a dummy variable for high polluting enterprises, Post = 1 when it is a high polluting enterprise, otherwise Post = 0. The estimation results are presented in column (4) of Table 2, which illustrates that the estimated coefficient did remains significantly negative at the 1% level when the Period*Post interaction term is included. After discharging for the green credit policy interference, similar results as the benchmark results are obtained.

### Analysis of impact mechanisms

The previous section confirms that the labor income share of firms decreases during the low-carbon transition process. Based on the theoretical part of the previous section, we argue that the carbon trading policy drives firms to increase productivity in the process of low-carbon transition, but the wage growth rate is not tandem with firms’ productivity, which decreases firms’ labor income share. This paper first explores whether carbon trading policies can increase in firm productivity. The semi-parametric method proposed by Olley and Pakes [31] and Levinsohn and Petrin [32], referred to as the OP method and LP method, are adopted to measure the firm’s total factor productivity and these two indicators are used to measure firm productivity levels. Regression of the did on total factor productivity is performed and the regression results are shown in columns (1) and (3) of Table 4. Notably, the regression coefficients are significantly positive at the 1% level, indicating that the carbon trading policy can promote the productivity level of enterprises. The results of regressing the firm productivity indicator on the firm labor income share are shown in column (2)(4) of Table 4, and the coefficients of lntfp_op and lntfp_lp are significantly negative at the 1% level, which is consistent with the findings by Zhou et al. [33] and Tong and Xu [34]. Further, to examine the impact of carbon trading policy on corporate profits, this paper regresses the did on corporate profits and obtains the results presented in column (5) of Table 4, which demonstrate that carbon trading policy increases corporate profits. Moreover, the firm’s profits on the labor income share are regressed and the results are shown in column (6) of Table 4, where an increase in firm profits reduces the labor income share. This means that an increase in labor productivity will increase the level of profitability of the firm, and the share of labor income will fall without increasing wage levels. Secondly, to test whether the carbon trading policy will decrease in the wage level of workers and thus decrease the share of labor income, this paper regresses did on the wage level of enterprises, where the wage level of enterprises is measured by the indicator of cash paid by enterprises for their employees in the current period. The results shown in column (7) of Table 5 suggest that the coefficient of did is significantly positive, indicating that the implementation of the carbon trading policy does not reduce the wage level of enterprises’ workers. Finally, the impact of carbon trading policy on the average wage level of enterprises is examined, where Meincome = cash paid by enterprises for employees in the current period / number of employees. The results are shown in column (8) of Table 4, reveal that the did coefficient is not significant, implying that the labor productivity and profit of enterprises increased successively after the implementation of carbon trading policy, but the income level of enterprise workers did not increase, leading to a decrease in the share of enterprise labor income.

**Table 4:** Analysis of impact mechanisms.

	(1)	(2)	(3)	(4)	(5)	(6)	(7)	(8)
VARIABLES	tfp_op	LS	lntfp_lp	LS	lnProfits	LS	lnincome	Meincome
did	0.057***		0.052***		0.100**		0.084**	0.035
	(3.85)		(3.91)		(2.49)		(2.51)	(0.98)
lntfp_op		-0.088***						
		(-32.90)						
lntfp_lp				-0.056***				
				(-20.99)				
lnProfits						-0.007***		
						(-11.20)		
Constant	-3.097***	0.394***	-4.041***	0.346***	2.675***	0.625***	0.466	6.288***
	(-7.59)	(12.93)	(-9.46)	(8.41)	(2.91)	(13.91)	(0.61)	(8.54)
Observations	16,539	16,539	14,435	14,435	15,856	15,856	17,142	17,124
Adjusted R-2	0.851	0.908	0.942	0.889	0.660	0.831	0.874	0.719
Individual fixed effects	Yes	Yes	Yes	Yes	Yes	Yes	Yes	Yes
Time Fix effect	Yes	Yes	Yes	Yes	Yes	Yes	Yes	Yes
Control variables	Yes	Yes	Yes	Yes	Yes	Yes	Yes	Yes

Note: Clustered robust standard errors are reported in parentheses below the coefficients.

***, **, and * denote significance at the 1%, 5%, and 10% levels, respectively. Control variables are estimated as in the Table 2 benchmark.

**Table 5:** Enterprise heterogeneity analysis.

	(1)	(2)	(3)	(4)	(5)	(6)
	Large scale	Small size	High Technology	Low Technology	High human capital	Low human capital
VARIABLES	LS	LS	LS	LS	LS	LS
did	-0.004**	-0.006	-0.006***	-0.002	-0.006***	-0.003
	(-2.55)	(-1.59)	(-3.11)	(-1.07)	(-3.08)	(-1.21)
Constant	0.671***	0.959***	0.673***	0.620***	0.647***	0.592***
	(9.72)	(11.77)	(12.62)	(7.16)	(12.43)	(6.42)
Observations	13,364	3,706	10,706	6,216	12,463	4,541
Adjusted R-2	0.843	0.790	0.833	0.820	0.792	0.872
Individual fixed effects	Yes	Yes	Yes	Yes	Yes	Yes
Time Fix effect	Yes	Yes	Yes	Yes	Yes	Yes
Control variables	Yes	Yes	Yes	Yes	Yes	Yes

Note: Clustered robust standard errors are reported in parentheses below the coefficients.

***, **, and * denote significance at the 1%, 5%, and 10% levels, respectively. Control variables are estimated as in the Table 2 benchmark.

### Heterogeneity analysis

To further examine the complexity of the impact of low-firm low-carbon transitions on labor income shares, this paper tests for differences in the impact of the policy on labor income shares across firm size, technology levels, etc.

#### Enterprise size

First, to determine whether differences in firm size under the carbon trading policy affect labor income shares, this paper divides firms into larger and smaller firms according to the median size of total assets and performs group regressions, and the results are presented in column (1)(2) of Table 5, where column (1) is the grouping of larger firms and the estimated coefficient of did is significantly positive. The estimated coefficient of did in column (2) is not significant, indicating that the carbon trading policy only affects the labor income share of larger firms and does not affect smaller firms.

#### Enterprise technology level

The determinant of firm productivity is the firm’s skill level. Therefore, based on the studies by Zhao et al. [35] and Xiao et al. [36], this paper divides the share of skilled workers into two groups of high-skilled level firms and low-skilled level firms, and group regressions are conducted separately, and the results are shown in columns (3)(4) of Table 5. The estimated coefficient of did is significantly negative for high skill level firms in column (3), while the estimated coefficient of did is not significant in column (4). This further demonstrates that the decline in the labor income share of firms is caused by the increase in the productivity level of firms.

#### Human capital

Since human capital is the source of technological R&D, corporate human capital determines the level of corporate R&D. Therefore, the impact of corporate human capital differences on the benchmark results should be investigated. For this purpose, we divide the sample into high human capital group and low human capital group based on the median of the percentage of graduate degrees and regresses them separately. The results are shown in column (5)(6) of Table 5, where column (5) has a significantly negative estimated coefficient of did in the high human capital group, while column (6) does not have a significant coefficient of did in the low human capital group. This implies that carbon trading policies cause reduce the share of labor income mainly for firms with higher levels of human capital. It further illustrates that the rise in productivity levels of firms after the implementation of carbon trading policy may not match the increase in labor income. Instead, firms with high human capital will provide higher compensation to highly skilled workers.

### Impact of firm entry and exit on labor income share

Finally, the impact of firm persistence status on labor income share is examined. Drawing on the approach of Li and Jiang [37], the firm status is classified into three categories: new entrants, incumbent firms, and exiting firms. Firms were defined as new entrants by determining whether they existed in the previous year, and if not, they were defined as exits in the following year; the firms that had been in the sample period were considered incumbents. The sample was divided into these 3 groups and regressed separately as shown in Table 6. Column (1) of Table 6 illustrates new entrants and the estimated coefficient of did is not significant. This is because, if high carbon enterprises enter the pilot region, they face higher costs, therefore, for rational enterprises, those with higher carbon emissions will choose non-pilot regions to enter, while low emission enterprises are more likely to enter the carbon trading pilot region. On the same note, new entrants will pay higher compensation to attract employees. In summary, carbon trading policies have less impact on labor productivity and employee compensation of new entrants, and hence have low impact on labor income share. Column (2) of Table 6 shows the sample of exiting firms and the estimated coefficient of did is insignificant. This is because, for exiting firms, which usually have lower productivity and higher carbon emissions, carbon trading policies have a high impact, which do not reduce carbon emissions through R&D, but choose to exit the market to face the cost shock of carbon trading policies. Therefore, carbon trading policies have a lower impact on the productivity of such firms, and thus a smaller change in the labor income share. Finally, the sample of incumbent firms are examined and the results are shown in column (3) of Table 6, which indicates that the estimated coefficient of did is significantly negative. This is because incumbent firms are more willing to face the carbon trading policy with a positive attitude, reducing carbon emissions and increasing firm productivity by improving technology and R&D. However, the growth of labor wages in such firms is much lower than the growth of firm productivity, resulting in a lower labor income share.

**Table 6:** Analysis of business status.

	(1)	(2)	(3)
	Enter	Withdrawal	Continuous
VARIABLES	LS	LS	LS
did	-0.001	0.032	-0.004**
	(-0.41)	(1.11)	(-2.31)
Constant	0.774***	0.917	0.629***
	(12.55)	(1.33)	(11.58)
Observations	5,585	167	13,859
Adjusted R-2	0.888	0.773	0.777
Individual fixed effects	Yes	Yes	Yes
Time Fix effect	Yes	Yes	Yes
Control variables	Yes	Yes	Yes

Note: Clustered robust standard errors are reported in parentheses below the coefficients.

***, **, and * denote significance at the 1%, 5%, and 10% levels, respectively. Control variables are estimated as in the Table 2 benchmark.

## Conclusion

Using the 2013 carbon trading policy pilot as a quasi-natural experiment, a DID identification framework is constructed in this paper to control for the endogeneity problem and empirically tests the impact of firms’ low carbon transition on their labor income share under the carbon trading policy. The following results are obtained: (1) Carbon trading policies can decrease the share of corporate labor income. (2) The implementation of the carbon trading policy has encouraged firms to increase their productivity during the low-carbon transition process, which indicates that the Porter hypothesis has been confirmed. However, productivity growth has not been in tandem with labor wage growth, leading to a decline in the share of labor income. (3) Carbon trading policy has a more significant impact on labor income of larger enterprises. (4) Given that the level of technology determines the productivity level of firms, carbon trading policies have a greater impact on the labor income share of firms with higher technology levels. (5) The carbon trading policy mainly "pushes" persistent incumbents to innovate and stimulate productivity improvement, and hence the carbon trading policy mainly affects the labor income share of persistent incumbents.

Based on the above conclusions, this paper draws the following insights. (1) The carbon trading policy can promote carbon emission reduction and motivate enterprises to improve total factor productivity, and the implementation of this policy will achieve a win-win situation in terms of economic and environmental benefits. Therefore, we should systematically and continuously promote adoption of the carbon emissions trading policy, provide more financial and taxation support to pilot regions with better implementation results, demonstrate the effect of pilot projects, and realize China’s carbon emission reduction commitments and high-quality and sustainable economic development in the new development stage. (2) To promote the low-carbon and green transformation of enterprises, we should pay more attention to the fairness of the initial distribution, improve the initial distribution mechanism to narrow the gap between the income distribution of capital and labor, establish a more reasonable income distribution pattern, and promote balanced social development.

There are some limitations in this paper. The data range used in this paper to investigate the impact of carbon trading policies on labor income share is from 2009 to 2016. The data span is relatively short and the data are relatively old.
